# From variant of unknown significance to actionable diagnosis: Stepwise interpretation of a novel *KRT2* variant in superficial epidermolytic ichthyosis with excellent retinoid response

**DOI:** 10.1016/j.jdin.2025.09.006

**Published:** 2025-10-01

**Authors:** Alexandra Carla Bobica, Sarah Moussa, Lydia Ouchene, Megha Udupa, Hessah BinJadeed, Rahaf Bashihab, Zuzanna Misiewicz, Laura Grönroos, Julie Powell, May Chergui, Tania Cruz Marino, William D. Foulkes, Elena Netchiporouk

**Affiliations:** a Division of Clinical and Translational Research, McGill University Health Centre, Montreal, Quebec, Canada; b Faculty of Medicine, McGill University, Montreal, Quebec, Canada; c Division of Dermatology, Department of Medicine, McGill University Health Centre, Montreal, Quebec, Canada; d Blueprint Genetics, Helsinki, Uusimaa, Finland; e Division of Dermatology, Centre Hospitalier Universitaire Sainte-Justine, Montreal, Quebec, Canada; f Department of Pathology, McGill University, Montreal, Quebec, Canada; g Department of Human Genetics, McGill University, Montreal, Quebec, Canada; h Division of Medical Genetics, Department of Specialized Medicine, McGill University Health Centre, Montreal, Quebec, Canada

**Keywords:** dermatopathology, epidermolysis bullosa, genetic counseling, genetics/genodermatoses, immunobullous, keratin, machine learning, retinoids

*To the Editor:* Superficial epidermolytic ichthyosis (SEI) is a rare autosomal dominant keratinization disorder caused by pathogenic variants (PVs) in *KRT2*, typically presenting with hyperkeratosis, trauma-induced blistering, and the Mauserung phenomenon.^1^ Although genetic testing enables definitive diagnosis in most inherited skin disorders, 30% to 60% of reports in dermatology identify a variant of uncertain significance (VUS), which can delay diagnosis and targeted treatment.^2^ Functional validation is often impractical, underscoring the need for systematic approaches that integrate phenotypic, histopathologic, and bioinformatics evidence for timely reclassification. We report a multigenerational family with a novel missense variant, *KRT2* c.566T>C (p.Phe189Ser), initially classified as a VUS and reclassified here as likely PV. Our objectives were to apply American College of Medical Genetics and Genomics/Association for Molecular Pathology criteria for reclassification,^3^^,^^4^ compare the family phenotype with published SEI cases, and evaluate systemic retinoid outcomes. We also provide a pragmatic reclassification algorithm for clinicians in Table I.

**Table I:** Practical framework for VUS interpretation in dermatology

Step	Action	Clear stop/proceed criteria	Example from SEI family case
1. Clinical assessment	Confirm if phenotype matches suspected genetic disorder. Document key cutaneous features, disease course, and atypical findings.	STOP if phenotype does not align—reclassification is not supported. PROCEED if phenotype supports the suspected condition (PP4 Supporting).	Proband showed classic SEI features: hyperkeratosis, blistering, and the Mauserung phenomenon.
2. Family testing and segregation analysis	Offer testing to affected/unaffected family members to evaluate variant segregation.	STOP if variant clearly does not segregate with disease—supports likely benign. PROCEED if segregation is supportive or unclear. Use PP1 (Supporting or Moderate∗) and combine with other evidence.	9 affected members all carried the KRT2 variant, supporting co-segregation (PP1 Moderate∗).
3. Population database review	Check population frequency in gnomAD, ExAC, etc.	STOP if variant frequency is >1% (suggests benign). PROCEED if variant is rare or absent (<0.01%) (PM2 Moderate).	Variant was absent from gnomAD, supporting rarity (PM2 Moderate).
4. Computational pathogenicity prediction	Use in silico tools like REVEL, PolyPhen-2, SIFT, and MutationTaster.†	STOP if REVEL ≥0.8 and ACMG criteria total ≥3 Moderate or ≥2 Moderate + ≥2 Supporting. PROCEED if REVEL <0.8 or criteria are unmet (PP3 Supporting).	REVEL score was 0.958, supporting PP3 (Supporting).
5. Compare to known pathogenic variants	Search ClinVar, HGMD, LOVD for same or similar changes.	STOP if the same residue/domain has a known pathogenic missense variant (PM5 Moderate) and the criteria threshold is met. PROCEED if data are absent or ambiguous.	A different missense variant (Phe189Tyr) at the same residue was classified as pathogenic (PM5 Moderate).
6. Expert consultation and functional confirmation	Refer to the genetics team or molecular board. Consider RNA/protein studies or modeling if needed.	STOP if expert consensus supports likely pathogenic classification per ACMG thresholds. PROCEED to functional studies if unresolved.	Multidisciplinary consensus supported reclassification without functional testing.
7. Patient counseling and follow-up	Explain implications of VUS. Guide monitoring and phenotype-based treatment. Plan reclassification or retesting as needed.	ALWAYS PERFORM this step. Clinical care should proceed based on phenotype, even without full reclassification.	Family members received phenotype-guided care and counseling on implications of a likely pathogenic variant.

Stop the VUS interpretation workflow only when: (1) ACMG/AMP criteria for “likely pathogenic” are met: ≥3 Moderate or ≥2 Moderate + ≥2 Supporting or expert consensus affirms reclassification.

*ACMG*, American College of Medical Genetics and Genomics; *AMP*, Association for Molecular Pathology; *CADD*, Combined Annotation Dependent Depletion; *ClinVar*, Clinical Variant Database; *ExAC*, Exome Aggregation Consortium; *gnomAD*, Genome Aggregation Database; *HGMD*, Human Gene Mutation Database; *LOVD*, Leiden Open Variation Database; *PM*, pathogenicity criterion—Moderate; *PP*, pathogenicity criterion—Supporting; *REVEL*, Rare Exome Variant Ensemble Learner; *SEI*, superficial epidermolytic ichthyosis; *SIFT*, Sorting Intolerant From Tolerant; *VUS*, variant of uncertain significance.

∗ PP1 is considered Supporting by default and can be upgraded to Moderate (if ≥5) or Strong (if ≥7) informative parent-to-child transmissions clearly show the variant co-segregates with disease. Each informative meiosis reflects a case where both genotype and phenotype are known and can be compared across generations (ACMG/AMP guidance).

† REVEL, PolyPhen-2, and SIFT are primarily used for missense variants. Tools like CADD and MutationTaster can be applied more broadly, including nonsense, splice-site, and noncoding variants. Use tools aligned with the variant type and interpret results in the context of other ACMG evidence.

This prospective cohort study was conducted at a multidisciplinary genodermatology clinic. Clinical evaluation, histopathology, next-generation sequencing, segregation analysis, and in silico prediction tools were used to determine variant pathogenicity. A scoping review summarized phenotypic variability in 67 genetically confirmed SEI cases. Retinoid outcomes were assessed retrospectively using the Subject Global Aesthetic Improvement Scale.^5^ The full methodology is detailed in the Supplementary, available via Mendeley at https://data.mendeley.com/datasets/9jbn4wznhs/1.

The proband (Fig 1, Patient IV-3 in Supplementary Fig 1, available via Mendeley at https://data.mendeley.com/datasets/9jbn4wznhs/1), a 27-year-old man, presented with longstanding flexural hyperkeratosis, recurrent blistering, and collarettes of peeling skin. Histopathology revealed epidermolytic hyperkeratosis with granular degeneration and suprabasal keratinocyte vacuolization. Targeted sequencing identified heterozygous *KRT2* c.566T>C and heterozygous *GJB2* c.101T>C (p.Met34Thr). Given the recessive inheritance of *GJB2*-related disease and normal audiometry, the *KRT2* variant was prioritized. Across 5 generations, 16 individuals exhibited SEI features; all 9 clinically affected members tested carried the *KRT2* variant, and none carried the *GJB2* variant, consistent with autosomal dominant inheritance (Supplementary Figs 1 and 2, available via Mendeley at https://data.mendeley.com/datasets/9jbn4wznhs/1). The *KRT2* c.566T>C variant was absent from gnomAD (PM2, Moderate), altered the same residue as a known PV p.Phe189Tyr (PM5, Moderate), and had high REVEL (0.958) and VEST-3 (0.977) scores (PP3, Supporting). It co-segregated with SEI in ≥5 informative meioses (PP1-Moderate) and matched the established phenotype (PP4, Supporting). Two Moderate and 4 Supporting criteria met American College of Medical Genetics and Genomics/Association for Molecular Pathology thresholds for “likely pathogenic.” Core features among affected relatives included hyperkeratosis (100%), blistering (66.7%), and the Mauserung phenomenon (88.9%). Palmoplantar keratoderma (11.1%) and hypertrichosis (33.3%) were less frequent. No erythroderma was observed. Phenotypic variability mirrored that in the scoping review (Supplementary Tables I and II, Figs 1 and 2, available via Mendeley at https://data.mendeley.com/datasets/9jbn4wznhs/1). Eight affected individuals received systemic retinoids (acitretin or isotretinoin) as part of routine care. All improved, with 5 of 8 (62.5%) achieving ≥75% improvement on the Subject Global Aesthetic Improvement Scale. Hyperkeratosis and skin peeling improved most consistently. Adverse events included mild mucocutaneous dryness (*n* = 3) and hypertriglyceridemia (4/5 isotretinoin-treated; none on acitretin) (Supplementary Table II, available via Mendeley at https://data.mendeley.com/datasets/9jbn4wznhs/1).

**Fig 1:**
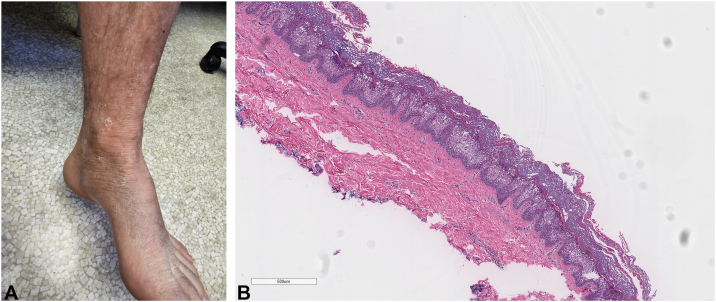
Clinical and histopathological features of superficial epidermolytic ichthyosis in the proband. **A,** Clinical image of the proband’s lower leg displaying marked hyperkeratosis and diffuse scaling, characteristic of SEI, observed before initiation of retinoid treatment. **B,** The histological section of the right wrist shave biopsy of the proband with SEI, stained with hematoxylin and eosin, showing characteristic features. The epidermis demonstrates hyperkeratosis with compact orthokeratosis and focal epidermolytic hyperkeratosis, as evidenced by eosinophilic granular degeneration and vacuolization of keratinocytes. The underlying dermis exhibits mild inflammation. *SEI*, Superficial epidermolytic ichthyosis.

This study demonstrates that a novel *KRT2* variant can be reclassified from VUS to likely pathogenic through integration of phenotype, segregation, and bioinformatics evidence, even without functional studies. SEI’s hallmark features can overlap with other keratinopathic ichthyoses and peeling skin syndromes, making molecular confirmation essential. Our findings support the use of systemic retinoids in SEI, although monitoring for adverse effects is critical.

By applying a reproducible workflow for variant interpretation, we enabled timely diagnosis, informed family counseling, and optimized patient care. This approach is applicable to other rare genodermatoses where functional assays are impractical but high-quality clinical, histopathologic, and computational evidence can guide reclassification.

## Conflicts of interest

None disclosed.
